# Network Analysis of Sexual Well-Being in Women with Heart Failure: The Psychocardiological Perspective

**DOI:** 10.3390/healthcare12080817

**Published:** 2024-04-11

**Authors:** Rafał Gerymski, Maria Latusek-Mierzwa

**Affiliations:** 1 Department of Health Psychology and Quality of Life, Institute of Psychology, Opole University, 45-040 Opole, Poland; 2 St. Hedwig’s Provincial Specialist Hospital, 45-221 Opole, Poland; maria.latusek-mierzwa@uni.opole.pl; 3 Doctoral School of the University of Opole, 45-040 Opole, Poland

**Keywords:** congestive heart failure, congestive cardiac failure, cardiovascular disease, women’s sexuality, sexual dysfunctions, perceived stress, anxiety, depression, fatigue

## Abstract

Sexuality is an important sphere of every person’s life. Sexual dysfunctions and sexual dissatisfaction may also be present in cardiac diseases. Individuals affected by heart failure (HF) deserve special attention since it can be the final stage of many cardiac diseases. Therefore, it is important to verify potential correlates of sexual well-being in individuals with HF. This study was conducted online between 2019 and 2023, and 262 Polish women aged between 18 and 59 years (*M* = 45.48; *SD* = 7.65) participated in it. The Short Sexual Well-Being Scale, Depression Anxiety and Stress Scale, Fatigue Assessment Scale, and authors’ questionnaire were used. Relationships between tested variables were verified with the use of network analysis performed with the *EBICglasso* estimator. Centrality assessment showed that sexual well-being had the highest values of betweenness, closeness and degree, followed by fatigue and depression measures. Sexual well-being was negatively related to the number of declared sexual dysfunctions, fatigue, stress and depression levels. Participants’ age and HF duration were not related to the sexual well-being of tested women. Multiple additional partial correlations were detected. The obtained results show that sexuality may be a central sphere of life in women with HF and that one’s sexuality should not be negated when working with cardiac patients.

## 1. Introduction

Heart failure (HF) is a very serious condition and is often the final stage of many cardiac diseases. It is most often caused by diseases of the cardiovascular system that affect the ejection of blood from and into the heart chambers. It occurs when the heart cannot pump enough blood to meet the body’s needs. Heart failure most often occurs as a result of chronic hypertension and chronic ischemic heart disease. Its symptoms most often include pulmonary and cardiological problems, such as breathing problems or swelling of the lower parts of the body. Patients diagnosed with HF get tired more quickly during physical exercise, which is often accompanied by heart palpitations. The most common psychological symptoms of the disease include depression, which is both a cause and a consequence of heart failure [1,2,3,4]. According to the Heart Failure Association (HFA) of the European Society of Cardiology (ESC), about 3 in 1000 individuals suffer from HF—both globally and in Poland [1,5]. What is more, women account for approximately half of the prevalent cases [6]. Heart failure is associated with many difficulties and its symptoms also significantly affect the daily functioning of people affected by it in the sexual sphere. Men with HF experience more sexual dysfunctions than women with the same diagnosis, but it is known that the level of sexual activity and libido decreases in both men and women. Studies indicate that nearly half of patients with heart failure reduce or completely stop sexual activity [7], which can negatively affect the well-being in HF patients [8].

Sexual well-being is a complex concept consisting of several factors, such as sexual health, physical pleasure, mental satisfaction and sexual distress, and is based on cognitive and emotional satisfaction with one’s sexuality [9,10,11,12,13]. It is important for every individual, as well as those suffering from somatic diseases and psychological problems [14,15,16,17,18,19]. Research shows that it also can play an important role in the functioning of cardiac patients. Altıok and Yılmaz’s [20] study on 232 cardiac patients shows that for 65.6% of respondents with heart disease, sex is one of the basic needs in life. What is more, 70% of their study participants claimed that heart disease has negatively affected their sexual lives. The respondents declared that the factors influencing the lower assessment of sexual life include fear of a heart attack during intercourse, problems with premature ejaculation and orgasm, reduced libido or old age [20]. It is known that sexual activity decreases in cardiac patients, like those after a heart attack (and especially among women), further affecting their sexual satisfaction [21,22].

As the number of HF symptoms and the severity of the disease increase, sexual activity also decreases or disappears, changing feelings of sexual pleasure and interest in the sexual sphere. All of this has a significant impact on the overall assessment of quality of life among people with heart failure [7]. Træen and Samara [23] show, in their large-scale study, that sexual dysfunctions are more frequent in cardiac patients than in healthy individuals. They also underline the fact that having sexual problems is a significant predictor of quality of life and sexual well-being among people with heart disease [23]. What is more, the fear of sexual intercourse associated with the disease’s progression can be related to the motivation to initiate sexual intercourse and its lower frequency [24,25]. HF symptoms (such as fatigue), its duration and progression can lead to increased levels of anxiety and depression symptoms, which play an important role in one’s quality of life and sexual sphere and often co-occur in people with heart failure [26,27,28,29,30,31,32,33,34]. Women are an important group in research on the sexual well-being of people with heart failure. Overall, women with heart failure have higher levels of depression and anxiety [26,31,34,35,36]. One of the factors co-occurring with depression in the group of people with heart disease is fatigue [28]. A higher level of depression is positively correlated with a higher level of fatigue among women with heart disease [26,34], and fatigue itself is related to the level of quality of life—the higher the level of fatigue, the lower the quality of life results in cardiac patients [27].

Based on the presented literature, it should be concluded that the sexual well-being in people with heart failure deserves crucial attention, both from the medical and psychological point of view. The study of stress, well-being and psychological functioning in cardiac disease is most often dealt with by a science called psychocardiology. Representatives of this field study the role of psychological and social factors in the development, course and treatment of cardiac diseases, as well as the processes of coping with heart diseases [37]. Based on the presented data, this study aimed to analyze the sexual well-being in heart failure patients and its correlates, such as age, HF duration, fatigue, depression, anxiety, stress and the number of sexual dysfunctions.

## 2. Materials and Methods

### 2.1. Participants and Procedure

This manuscript presents the results of the cross-sectional study, which was performed online. This study was designed and carried out in accordance with the guidelines of the Bioethics Committee at the Institute of Medical Sciences of Opole University (guided by the Act of 5 December 1996 on Professions Of Doctor And Dentist, issued by the Polish Sejm—the lower house of the Parliament of Poland) and in line with the Declaration of Helsinki’s ethical principles.

The presented results were collected online between 2019 and 2023. Study participants were accessed via social media platforms related to cardiac diseases and heart failure. This method of recruitment was used to provide the respondents with a sense of security and anonymity of their data, which is especially important when dealing with such a sensitive topic as sexuality. All participants were informed about the anonymity of the obtained results and that they had the possibility of stopping participation in the study at any time. The presented study is part of the ongoing project focused on cardiac health conducted by the first author (R.G.). It received a positive recommendation from the Bioethics Committee at the Institute of Medical Sciences of Opole University (application numbers: 2/KB/12/2019). According to the committee’s decision, cross-sectional studies do not raise any ethical concerns.

This study failed to recruit a sufficient number of men, which would allow the authors to perform a network analysis. For this reason, their results were excluded from the analysis. In total, 262 women with a HF diagnosis took part in this study. The inclusion criteria were as follows: (1) being an adult (18 years and above); (2) without cognitive deficits; (3) having with ability to make independent decisions; (4) whose native language was Polish; (5) suffering from heart failure; (6) and having an NYHA scale score of 2 or 3. The exclusion criteria included the following: (1) being hospitalized in the past 6 months; (2) having an NYHA score of 1 (no heart failure) or 4 (significant heart failure, severely impairing functioning). The above information was verified based on the respondents’ declarations.

### 2.2. Measures

Sociodemographic and disease-related data were measured using the authors’ questionnaire consisting of many structured and open-field questions, related to the participants’ gender, place of residence, marital status, education, employment, lifestyle, diagnosis history and prescribed pharmacotherapy (see Table 1 for more detailed information). This study also used three psychometrically validated measures: the Short Sexual Well-Being Scale, Depression Anxiety and Stress Scale, and Fatigue Assessment Scale.

Sexual well-being was measured with the Short Sexual Well-Being Scale (SSWBS) [10]. It contains 5 items such as “I consider myself sexually fulfilled” on a 7-point answer scale (1—I completely disagree; 7—I completely agree). A higher score indicates higher sexual well-being. In the present study, the SSWBS had good psychometric properties (Cronbach’s *alpha* = 0.94; McDonalds’ total *omega* = 0.95; *CFI* = 0.98; *TLI* = 0.97; *SRMR* = 0.03; *RMSEA* = 0.10).

Depression, anxiety and stress symptoms were measured using DASS-21 [38]. It contains 21 items such as “I felt I was close to panic” on a 4-point scale (0—Did not apply to me at all; 3—Applied to me very much or most of the time). DASS-21 allows for the calculation of 3 subscales regarding the level of perceived severity of depression, anxiety and stress. A higher score in each subscale means greater symptom severity. The Polish version of DASS-21 was corrected for this study because the available Polish adaptations contained semantic and grammatical errors. Therefore, its psychometric properties were tested. In this study, the scale demonstrated acceptable psychometric properties (Cronbach’s *alpha* = 0.95; McDonalds’ total *omega* = 0.96; *CFI* = 0.94; *TLI* = 0.91; *RMSEA* = 0.15).

Fatigue was measured using the Fatigue Assessment Scale (FAS) [39]. It contains 10 items, such as “Mentally, I feel exhausted” or “I get tired very quickly”, related to the levels of physical and mental fatigue. According to the original validation of the scale, the summary score reflecting overall fatigue had the best psychometric properties. Therefore, only the summary score of FAS was used for this study. FAS items can be answered on a 5-point answer scale (1—Never; 5—Always). The authors of the original version of the FAS consented to the Polish translation of the scale via e-mail correspondence. In this study, it obtained good reliability and model fit coefficients (Cronbach’s *alpha* = 0.81; McDonalds’ total *omega* = 0.84; *CFI* = 0.95; *TLI* = 0.92; *RMSEA* = 0.09).

### 2.3. Statistical Analysis

Relationships between tested variables were verified with the use of network analysis. Before performing the statistical analysis, we decided to verify whether or not the obtained sample of HF patients allowed for calculating the network. Unfortunately, at the moment, there are no reliable methods to perform power analysis for the tested network models. One researchers’ proposal based on the Monte Carlo simulation is based on arbitrarily selected tuning parameters [40]. As the values of those tuning parameters are not justified, this method of sample size calculation cannot be considered valid. Therefore, we decided to verify the size of the obtained sample based on the rule-of-thumb methods. Many recommendations note that a sample size of 250 people is recommended for calculating small networks [41,42]. On this basis, the obtained sample of women with HF was considered sufficient for the proposed analysis.

Most of the tested variables included in Table 1 indicated very homogeneous characteristics of the studied group. Moreover, variables with bigger variance in responses did not show adequate numbers in subgroups to be included in the analysis. On this basis and given the size of the studied sample, a network analysis was performed using only the following variables: the studied women’s age, HF duration, levels of fatigue, depression, anxiety and stress, number of declared sexual dysfunctions and levels of sexual well-being.

The network model was calculated using the *EBICglasso* estimator, based on the partial correlation coefficients. Partial correlation coefficients reflect the relationship between two variables after controlling for all other tested relationships, which makes them similar to the standardized coefficients obtained in the multiple regression analysis. The partial correlation network was also used because it maps out multicollinearity and limits the number of spurious connections by setting very low relationships equal to zero [42,43,44]. The network analysis was calculated in JASP 0.11.1.0 statistical software [45]. The following tuning parameters for network analysis were used: γ (*gamma*) = 0.50 [42,43,44], and JASP’s default λ (*lambda*). Based on the recommendations [42,43,44,46,47,48], network centrality was also analyzed. To further verify the correctness of the obtained network model, edge and centrality stability analysis was performed. Edge stability was verified with the usage of non-parametric bootstrapping with the declared number of 1000 samples [43]. It allows the calculation of confidence intervals, where width reflects the accuracy of parameter estimates [46]. When the bootstrapped confidence intervals are wide, it becomes hard to interpret the strength of the edge [42]. The network’s centrality was verified with the use of subset bootstrapping. It simulates how centrality indices behave after dropping a particular % of the cases presented in the dataset. In this visualization, the y-axis represents the values of the centrality stability coefficient (*CS*)—which reflects the correlation between the original centrality indices and those obtained by dropping a particular % of study participants. It is suggested that the *CS* coefficient should be equal to 0.70, and after dropping many cases, it should be above 0.50 [42].

## 3. Results

### 3.1. Sociodemographic Characteristics of the Studied Sample

In total, 262 women aged between 18 and 59 years (*M* = 45.48; *SD* = 7.65) participated in this study. A typical research participant could be characterized as a middle-aged woman, living in a town (over 68% of respondents), married (over 62%), with higher education (over 64%), and working (over 80%). The exact characteristics of the studied sample are presented in Table 1.

### 3.2. Network Edge Analysis

Table 2 presents the values of the edge weights obtained in the network analysis using the *EBICglasso* estimator. The analysis showed that sexual well-being was moderately and negatively related to the levels of fatigue and the number of declared sexual dysfunctions. What is more, the results of the SSWBS scale were weakly and negatively related to the DASS-21 scores. This indicated that the higher the fatigue, number of sexual dysfunctions, depression, anxiety and stress, the lower the sexual well-being. What is more, depression levels were strongly and positively associated with the declared severity of anxiety, and weakly with perceived stress. The analysis also showed a weak but positive relationship between fatigue and stress—the higher the levels of fatigue, the higher the levels of perceived stress. Additionally, a moderate and positive relationship between participants’ age and HF duration was observed. Overall, the obtained network model can be considered sparse, with few relationships between nodes. See Table 2 for more detailed results.

**Figure 1:**
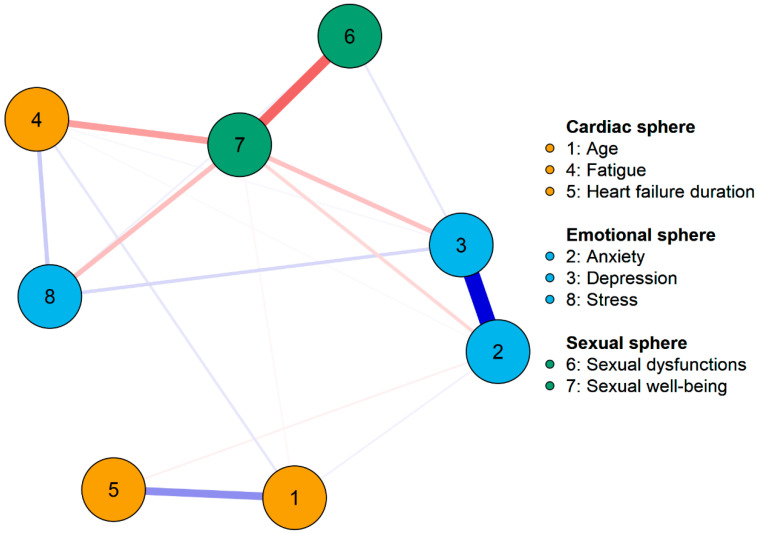
The obtained network model.

Figure 1 shows the visualization of the obtained model. It can be seen that the tested variables form some specific clusters. Variables related to the sexual sphere are located close to each other. Anxiety and depression also form a strongly related cluster. Age and HF duration form a cluster distant from the central part of the model and are not significantly related to any of the other variables (the relationship between age and fatigue is very weak). The visualization of the model introduces some suspicions about clusters and potential mediating relationships, but unfortunately, the obtained sample size does not allow for a more detailed examination of these relationships. Therefore, in the discussion of the results, only the relationships between the tested variables (their strength and direction) will be analyzed, and not the model’s cluster structure. We encourage readers to use the structure presented in Figure 1 only as additional guidance in reading the data presented in Table 2.

### 3.3. Network Centrality Analysis

**Figure 2:**
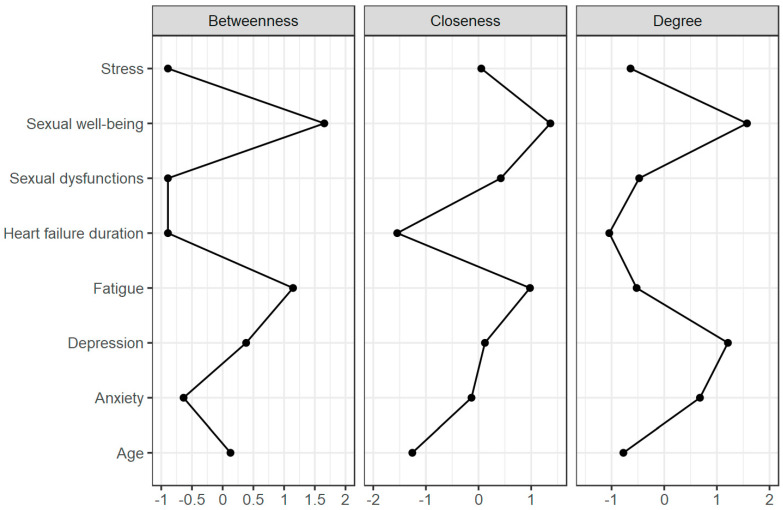
The obtained centrality plot.

Centrality assessment showed that sexual well-being had the highest values of betweenness, closeness and degree, followed by fatigue and depression measures. Therefore it can be concluded that changes in those variables could have the biggest impact on the whole model. The obtained centrality plot is presented in Figure 2.

### 3.4. Edge and Centrality Stability Analysis

**Figure 3:**
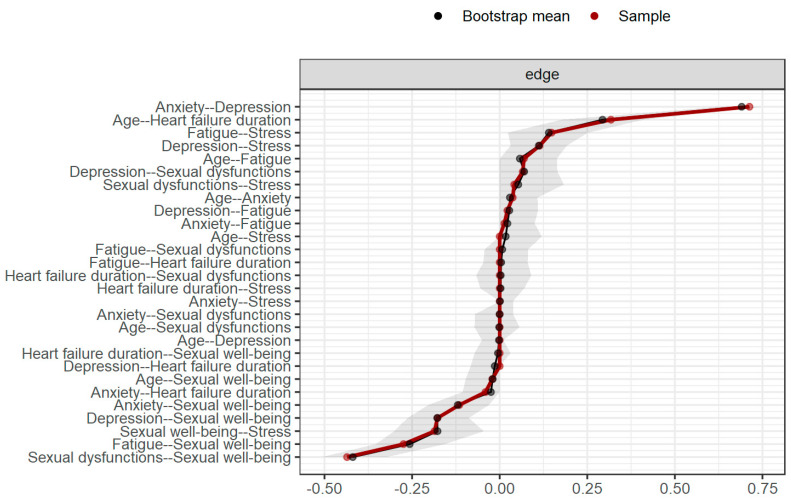
Results of the edge stability analysis.

To further verify the correctness of the obtained network model, edge and centrality stability analysis was performed. The obtained results are shown in Figure 3. It can be concluded that some of the presented confidence intervals can be considered wide. This is probably related to the fact that the sample size is acceptable but still fairly low. Therefore, it is suggested that readers interpret the results of edge weights with caution.

**Figure 4:**
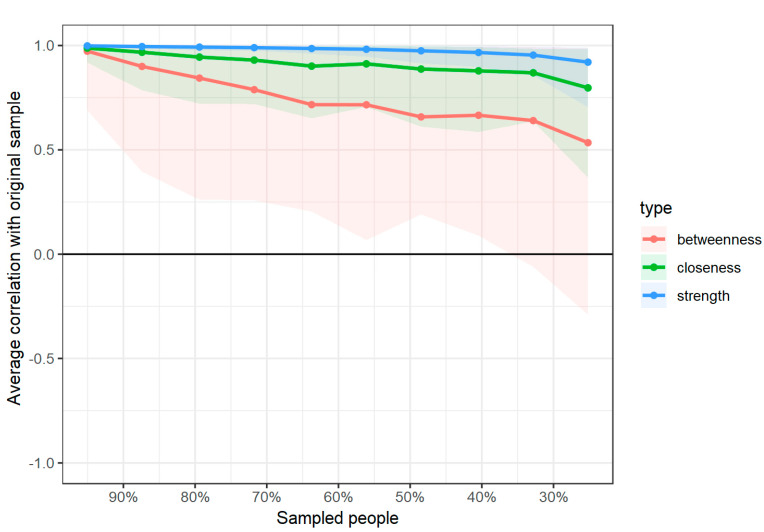
Results of the centrality stability analysis.

Lastly, subset bootstrapping was performed to verify the stability of the network’s centrality. The results are shown in Figure 4. The performed analysis shows that despite the sample size being fairly small, even after dropping 70% of the cases, the *CS* coefficient exceeds the recommended value of 0.50. Therefore, it can be concluded that the obtained model’s centrality was stable (see Figure 4).

## 4. Discussion

Sexual well-being is an important factor for women with heart disease. It plays an important role in the overall level of quality of life of cardiac patients [20,49]. It is known that women who declare a deterioration in their sexual satisfaction due to heart disease have a lower level of quality of life [8]. In the presented study, it was verified that sexual well-being was significantly related to the levels of sexual dysfunction, fatigue, depression, anxiety and stress. Therefore, it can be concluded that functioning in the sexual sphere is an important factor contributing to the functioning of women with heart failure.

In the presented study, sexual well-being was most moderately associated with the occurrence of sexual dysfunctions and fatigue. It was confirmed before that the presence of sexual dysfunctions can also negatively affect sexual satisfaction in cardiac patients [20]. Therefore, the results of the presented study are not surprising. They also indicated that fatigue was the second most important factor related to sexual well-being in a group of women with heart failure. Research shows that a higher level of fatigue is characteristic of women with heart diseases [26]. Poor physical and emotional well-being (which can be manifested by fatigue) may significantly affect sexual well-being. It may be assumed that physical and emotional symptoms of fatigue discourage engagement in sex life or make sexual intercourse impossible, therefore reducing their frequency and satisfaction.

The results of this study additionally showed that stress, depression and anxiety were also important factors associated with sexual well-being in a group of women with heart failure. It is important to note that the obtained relationship coefficients indicate weak relationships between sexual well-being and variables related to the psychological functioning of the studied group. Studies show that the severity of symptoms of depression and anxiety can be related to the reduced level of the overall quality of life [29,31,32,33,50,51]. Sexual well-being is an important component of the overall quality of life, and anxiety and depression can be associated with its reduced level [49]. The levels of anxiety may be related to sexual intercourse itself, because people with heart filature may be afraid that the symptoms of the disease may increase during sex, or that they may have a heart attack during sexual intercourse [20]. However, the weak correlations of depression, anxiety and stress with sexual well-being observed in this study might be related to the fact that other studied factors, such as the occurrence of sexual dysfunctions and fatigue, might have a greater impact on sexual well-being because they are related to both physical and emotional spheres of well-being.

In this study, age was not related to the sexual well-being and emotional sphere of the studied women. This is consistent with the results of research on depression and anxiety among people with heart failure, which showed that they were related to the occurrence and type of cardiac disease, rather than the age of the studied patients [50]. Also, the duration of heart failure was not significantly related to any studied variables other than anxiety (a very weak relationship). It can be assumed that the length of the disease was not significantly related to the sexual well-being of the studied women with heart failure because the duration of the disease is probably less important than its severity—the frequency and of its symptoms and how bothersome they are [4,26,27,28,29,30,31,32,33], which can be also observed in this study in the relationship between fatigue and sexual well-being.

No scientific research is without limitations. The described project also has them. First, the presented study was cross-sectional. Additional longitudinal studies should be conducted to confirm the validity of the obtained results. It is important to also perform replication studies of the presented project due to the fact that sexual well-being was a central element of the model obtained in network analysis, but this may have been due to the subject of the study, which could have significantly influenced the respondents’ answers. Second, the tested sample is not representative; it did not include the results of men and its size does not correspond to the population of people suffering from HF in Poland. Nationwide research on the sexuality of people with HF should be conducted to confirm the correctness of the presented results. Thirdly, subsequent research should include measurements of sexual well-being and quality of life. Despite the obvious connection between the sexual sphere and the general quality of life of every person, it is necessary to specifically verify the strength of this relationship in the examined sample of people. Fourth, the study measured stress using DASS-21, which asks about the events over the past 7 days. It would be more beneficial to examine chronic stress using tools such as the Trier Inventory for Chronic Stress [52] or Chronic Stress Scale [53]. Lastly, due to the nature of the study (an online study with information based on declarations of respondents, not supported by medical data), it did not contain biomedical data on the characteristics of the disease and its course in the study participants, such as blood pressure, HDL and LDL cholesterol levels, blood sugar levels, the specific type and characteristics of heart failure, etc. Further Polish research should be conducted in cooperation between psychologists and cardiologists to exchange important biomedical and psychological information, which would allow researchers to explain the obtained results more accurately and in greater detail.

Despite its limitations, the presented study has important practical implications, both for psychologists and cardiologists. It underlines the fact that the sexuality of women with heart failure can act as a central sphere of psychological functioning in those patients. Therefore, when working with such a group of cardiac patients, specialists should also focus their support efforts on sexual well-being and possible dysfunctions in heart failure patients. Unfortunately, detailed information about sexual life is not part of the clinical interview during hospital admission in Poland [18]. This study is one of the first to emphasize the importance of sexual well-being in the lives of Polish cardiac patients. Cardiovascular diseases have significant limitations that may make it impossible to lead a satisfactory sex life. Therefore, specialists working with people with HF should try to teach patients to enjoy their sexuality, despite its limitations. This psychoeducation can be performed based on the “Good-Enough Sex” model by Metz and McCarthy [54]. It involves teaching patients that sex life has its ups and downs—that sometimes it is very good, and sometimes it is dysfunctional. Working with patients in the light of this model can show them how to derive satisfaction and other values important for relationships despite the difficulties in meeting sexual needs.

## 5. Conclusions

Heart failure is an event that significantly affects the quality of life and sexual well-being of all patients diagnosed with it. Patients who suffer from heart failure show reduced sexual well-being. This may be related to physical symptoms of the disease, such as fatigue, but the psychological factors also appear to be involved in the decreased sexual well-being of people with heart failure. In the group of women with heart failure, psychological factors such as depression and anxiety were associated with reduced sexual well-being. Poorer physical and psychological functioning may lead to decreased sexual activity or less satisfaction from one’s sexuality. This decreased sexual activity may be also related to the fear of severe symptoms of heart failure during sexual intercourse, fear of a heart attack or general decreased in libido, which can be a side effect of the medications taken. Higher sexual well-being is associated with higher overall well-being, so paying attention to this area of life in women suffering from heart failure is necessary.

## Figures and Tables

**Table 1:** Characteristics of the studied sample of heart failure patients—information declared by the study participants and unsupported by medical records (*N* = 262).

	*M*	*SD*	*Me*	*Min*	*Max*
Age	45.48	7.65	46.00	18.00	59.00
Heart failure duration	3.54	1.94	3.00	1.00	7.00
Number of sexual dysfunctions	1.13	1.20	1.00	0.00	3.00
				*n*	%
Gender					
	Women			262	100.00%
	Men			0	0.00%
Place of residence					
	Town/city			179	68.32%
	Village			83	31.68%
Marital status					
	Married			163	62.21%
	Informal relationship			91	34.73%
	Single			8	3.05%
Education					
	ISCED 6–8: Tetriary: Bachelors and higher (in Poland: *wyższe*)			170	64.89%
	ISCED 3: Upper Secondary: High School (in Poland: *średnie*)			92	35.11%
Employment status					
	Employed			212	80.92%
	Unemployed			50	19.08%
Lifestyle information					
	Alcohol consumption			207	79.01%
	Smoking tobacco cigarettes			31	11.83%
	Smoking e-cigarettes			21	8.02%
	Physical activity—swimming			17	6.49%
	Physical activity—bicycle riding			11	4.20%
	Physical activity—walking			49	18.70%
	Being under the care of a cardiology clinic			195	74.43%
	Being under the care of a psychologist			25	9.54%
	Being under the care of a psychiatrist			7	2.67%
	Being hospitalized in the past 6 months			0	0.00%
Cardiological diagnosis					
	Heart failure (NYHA II & III)			262	100.00%
	Coronary heart disease			104	39.69%
	Primary hypertension			233	88.93%
	Secondary hypertension			9	3.44%
	Myocardial infarction			3	1.15%
	Type 1 diabetes			8	3.05%
	Type 2 diabetes			22	8.40%
	Atherosclerosis			48	18.32%
Other diseases and illnesses					
	Depression			23	8.78%
	Hypothyroidism			19	7.25%
	Psoriasis			4	1.53%
	Insulin resistance			1	0.38%
Implanted devices					
	Cardiac resynchronization therapy (CRT)			1	0.38%
	Implantable cardioverter–defibrillator (ICD)			3	1.15%
Pharmacotherapy *					
	Metoprolol			21	8.02%
	Spironolactone			17	6.49%
	Perindopril			13	4.96%

Note: * Other (declared by less than 10 study participants): amlodipine, apixaban, atorvastatin, candesartan, eplerenone, flecainide, furosemide, lercanidipine, metformin, propranolol, ramipril, sacubitril and valsartan combination, torasemide, or warfarin. All of the presented information was declared by the study participants and is unsupported by medical records due to the lack of access to such documentation.

**Table 2:** Edge weights obtained in the network analysis using the *EBICglasso* estimator (*N* = 262).

		*k* of Nodes		*k* of Non-Zero Edges		Sparsity	
		8		17/28		0.393	
	Age	HF Duration	Fatigue	Depression	Anxiety	Stress	Sexual Dysfunctions
Age	-						
HF duration	0.317	-					
Fatigue	0.069	-	-				
Depression	-	-	0.022	-			
Anxiety	0.037	−0.041	0.013	0.713	-		
Stress	-	-	0.148	0.114	-	-	
Sexual dysfunctions	-	-	-	0.066	-	0.041	-
Sexual well-being	−0.021	-	−0.275	−0.177	−0.115	−0.186	−0.436

Note: the edge weights equal to zero were removed from the table to improve its readability.

## Data Availability

The data can be made available from the corresponding author upon reasonable request.
